# Temporomandibular Disorders and Orofacial Outcomes in Subjects with Neck Pain and/or Cervicogenic Headache: A Systematic Review with Meta-Analysis

**DOI:** 10.3390/jcm15010266

**Published:** 2025-12-29

**Authors:** Paolo Bizzarri, Andrea Giusti, Marco Pernici, Paolo Bulzacca, Giacomo Asquini, Filippo Maselli, Firas Mourad, Edoardo Balli, Giulia Pisacane, Cecilia Bagnoli, Anna Manzari, Marco Pompi, Aldo Scafoglieri

**Affiliations:** 1 Experimental Anatomy Research Group (EXAN), Vrije Universiteit Brussel (VUB), B-1090 Brussels, Belgium; 2 Department of Human Neurosciences, University of Roma “La Sapienza”, 00185 Rome, Italybulzaccafisio@gmail.com (P.B.); masellifilippo76@gmail.com (F.M.); manzari.anna15@gmail.com (A.M.);; 3 Centre of Precision Rehabilitation for Spinal Pain, School of Sport, Exercise and Rehabilitation Sciences, College of Life and Environmental Sciences, University of Birmingham, Birmingham B15 2TT, UK; 4 Department of Health, LUNEX University of Applied Sciences, 50, Avenue du Parc des Sports, 4671 Differdange, Luxembourg; 5 Luxembourg Health & Sport Sciences Research Institute A.s.b.l., 50, Avenue du Parc des Sports, 4671 Differdange, Luxembourg; 6 Facoltà Dipartimentale di Medicina e Chirurgia, Università Campus Bio-Medico di Roma, 00128 Roma, Italy

**Keywords:** temporomandibular disorders, headache, neck pain, cervical pain, cervicogenic headache, meta-analysis

## Abstract

**Introduction**: Temporomandibular disorders (TMDs), neck pain (NP), and cervicogenic headache (CGH) frequently co-occur. We aimed to assess TMD prevalence and orofacial clinical features in adults with NP or CGH versus asymptomatic controls. **Methods**: We searched PubMed, CINAHL, Web of Science, and Scopus from inception to 31 July 2025. Eligible designs were analytical cross-sectional studies comparing TMD prevalence, signs, or symptoms between NP/CGH patients and controls. Outcomes included TMD prevalence, jaw mobility, masticatory muscle pressure pain thresholds (PPT), and palpation findings. Risk of bias was appraised with the JBI analytical cross-sectional checklist. Random-effects meta-analyses synthesized odds ratios (ORs) for dichotomous and mean/standardized mean differences (MDs/SMDs) for continuous outcomes; heterogeneity was quantified with I^2^ (and τ^2^ where available). Small-study effects were inspected visually (k < 10). Certainty of evidence was assessed with GRADE. **Results**: From 4130 records, nine studies met the criteria (eight NP, 400 subjects; one CGH, 44 subjects). NP was associated with higher TMD prevalence versus controls (OR 3.64, 95% CI 1.35–9.84; I^2^ = 13%). Jaw mobility was reduced in either pain-free opening (one study), unassisted opening (one study), or maximum assisted opening (three studies; MD −6.16 mm, 95% CI −10.05; −2.28; I^2^ = 83%). PPTs were lower in symptomatic groups for masseter (SMD −1.11, 95% CI −1.89 to −0.32; three studies; I^2^ = 92.6%) and temporalis (SMD −0.77, 95% CI −1.04 to −0.50; five studies; I^2^ = 69%). Myofascial trigger points and pain on palpation of masticatory muscles or TMJ were more frequent in experimental groups. **Discussion**: The findings suggest consistent associations between NP/CGH and TMD prevalence with signs of orofacial dysfunctions. Certainty of evidence was very low due to the cross-sectional design, incomplete confounding control, and moderate heterogeneity for several outcomes. **Conclusions**: Adults with NP/CGH show higher TMD prevalence and reduced jaw mobility with lower masticatory PPTs. The results support integrated assessment, and prospective longitudinal studies are needed.

## 1. Introduction

Temporomandibular disorders (TMDs) are a heterogeneous group of musculoskeletal conditions involving the temporomandibular joints (TMJs), masticatory muscles, and associated orofacial structures [1]. Primary characteristics can include pain in the jaw area, restricted mouth mobility, and TMJ sounds. The Research Diagnostic Criteria for Temporomandibular Disorders (RDC/TMD) [2] and the more recent Diagnostic Criteria for Temporomandibular Disorders (DC/TMD) [1] are internationally recognized reference standards for diagnostic classification. Patients with TMDs often report symptoms of neck pain or headache [3].

Neck pain (NP), particularly in its persistent stage, constitutes an extremely prevalent musculoskeletal condition [4]. In the United States, a prevalence of 54% has been estimated in a population sample of 189,977 subjects [3,5], with higher prevalence in women. Cervicogenic headache (CGH) is a specific headache subtype, recognized as a secondary headache linked to the cervical region, classified by the International Headache Society (IHS) and included in the International Classification of Headache Disorders (ICHD-3) [6]. Diagnosis of CGH relies on specific characteristics of CGH, including reduced neck mobility, mechanical pain provocation, ipsilateral neck and arm pain, and relief via local anesthetic blocks. Although less common, it has an estimated prevalence of 0.4–2.5% in the general population, and 15–20% in patients with chronic headache [7].

Signs and symptoms of TMDs are common in the general population, with prevalence rates varying considerably across studies depending on the diagnostic criteria used [8]. Most reports converge on an estimated prevalence of approximately 10–15%. A diagnosis of joint-related TMDs (e.g., disc displacement with reduction) does not necessarily imply the need for treatment, which should be guided by the presence of pain or functional limitation and considered within a biopsychosocial framework [9].

Despite being distinct clinical entities, TMDs, cervical pain, and headache are frequently reported as coexisting conditions, posing significant challenges in clinical management. Several studies have shown a high prevalence of NP and/or headache in TMD patients. Specifically, the prevalences of headache and NP in patients with TMDs have been reported at 54.2% (OR. 7.0; 95% CI 6.6–7.5) and 52.5 (OR 7.9; 95% CI 7.5–8–4), respectively [3]. Specifically, CGH has been reported in 28% of patients with TMDs [7,10]. Moreover, patients with migraine and tension-type headache have an overall higher risk of TMDs, with an increased prevalence associated with higher headache frequency [11].

To explain the comorbidity between TMDs, NP, and CGH, numerous mechanisms have been hypothesized highlighting a complex neurophysiological and biomechanical interrelationship. The convergence of proprioceptive and nociceptive afferents from the C1–C4 into the trigeminal nucleus caudalis suggests a reciprocal interaction under dysfunctional conditions, providing a neurophysiological basis for clinical overlap [12]. This trigeminocervical convergence may facilitate referred pain between the two regions, associated with a regional mechanical hypersensitivity (e.g., myofascial pain). Central and peripheral sensitization are believed to play a crucial role, with patients experiencing concomitant chronic neck pain (CNP) and myogenic TMDs often demonstrating more widespread pain and distal hyperalgesia, fostering chronic pain conditions [13]. Furthermore, masticatory muscle pain is frequently observed in individuals with neck and shoulder disorders, including whiplash-associated disorders [14].

TMJs are closely related to the cervical region from an anatomical and biomechanical point of view. Although the scientific literature does not support a postural influence of TMDs and orofacial pain on spinal pain [15,16], a biomechanical interrelationship between the jaw and the cervical spine exists. Experimental studies showed an influence of artificial positioning of the cervical spine on TMJ biomechanics [17]. In addition, specific activities of the jaw are reported to modify the excursion of the Flexion Rotation Test [18]. Awake and sleep bruxism may represent key factors in masticatory muscle overload, particularly jaw bracing [19]. Such bruxism behaviors have been shown to involve the activation of cervical muscles [20], potentially facilitating NP.

Subjects with chronic craniofacial pain often report a disabling condition, linked to psychological distress. Perceived stress, anxiety, and depression may represent a link between bruxism, TMDs, headaches, and NP. Collectively, these factors can enhance central mechanisms of pain perception, therefore promoting symptom spreading, and have been found to be important risk factors for TMDs or NP and their chronicity [21,22].

Although extensive literature has examined the prevalence and severity of NP and headache in individuals with TMDs, and a significant clinical association has been established [11,16], to the best of our knowledge, to date, no systematic review has directly compared TMD diagnoses and orofacial signs and symptoms in patients with NP and/or CGH against asymptomatic controls. Identifying these associations is crucial for a better understanding of the complex interaction of these conditions and could guide diagnostic and therapeutic management, even in patients who do not present with apparent facial pain but may show pre-clinical signs of TMDs, thereby facilitating early and more effective interventions. Several studies have examined temporomandibular disorder-directed interventions (e.g., physiotherapy, arthrocentesis) and report measurable effects on neck pain in patients with these comorbid conditions [23].

Therefore, the objective of this systematic review was to synthesize the available evidence from cross-sectional studies to evaluate and compare the presence and characteris-tics of TMDs and orofacial outcomes in individuals affected by cervical pain and/or CGH compared to healthy subjects.

## 2. Materials and Methods

This systematic review was conducted in accordance with the guidelines of the Meta-analysis of Observational Studies in Epidemiology (MOOSE) [24] group and the Preferred Reporting Items for Systematic Reviews and Meta-Analyses (PRISMA) [25] (Table S4). The protocol was prospectively registered in the PROSPERO database (registration number: CRD420250621942).

### 2.1. Information Sources

We included analytical cross-sectional studies published in English that compared the presence and characteristics of TMDs in adults with NP and/or CGH to adults without such disorders. A comprehensive electronic search was performed in PubMed, CINAHL, Web of Science, and Scopus. The literature search was performed from inception until 7 July 2025. The complete search strategy is available in the Supplementary Material (Table S3).

No time restrictions were applied to the searches. Additionally, reference lists of the included studies were screened for further eligible articles. All screening was performed using the Rayyan platform (https://www.rayyan.ai/; accessed on 10 July 2025). Our preliminary search yielded no longitudinal observational studies (e.g., cohort or retrospective designs) investigating the association between CGH or NP and TMDs or other orofacial outcomes. Accordingly, the present systematic review was restricted to cross-sectional studies.

### 2.2. Eligibility Criteria

Eligible studies were required to meet the following criteria:

- Design: cross-sectional studies;
- Adult population (≥18 years);
- Exposure group: subjects with cervical pain and/or CGH, defined according to the International Classification of Headache Disorders (ICHD-3) or authors’ diagnostic criteria consistent with these definitions;
- Control group: subjects without cervical pain or CGH;
- Outcomes: prevalence of TMD, or assessment of TMD-related signs and symptoms (e.g., bruxism, orofacial pressure pain thresholds (PPTs), myofascial trigger points, and mandibular mobility).

Exclusion criteria included

- Case reports;
- Studies on adolescents or pediatric populations;
- Studies lacking a control group;
- Headache or orofacial symptoms without a clear diagnostic framework (e.g., RDC/TMD, ICHD-3).

Studies limited to headache or reporting only non-specific “facial pain” were excluded to avoid diagnostic overlap with odontogenic pain, trigeminal neuralgia, or primary headache disorders [26]. Studies including experimental groups with combined craniocervical comorbidities (e.g., neck pain with TMDs or neck pain with primary headaches) were excluded. Given the heterogeneity of TMD classifications across the literature, we accepted diagnostic criteria as reported in each study if they referred to validated clinical tools (e.g., RDC/TMD, DC/TMD).

When available, subgroup analyses were planned according to the specific TMD diagnosis. In studies that reported both joint and painful TMDs, data were extracted separately for each subgroup. Conversely, in studies where the two conditions were reported as mutually exclusive, and only one diagnosis was provided, the TMD was classified as “unspecified”. Meta-regression was conducted when ≥10 studies contributed to the outcome [27]; otherwise, heterogeneity was addressed via planned subgroup or narrative synthesis.

In studies comparing multiple groups, data extraction was limited to participants presenting with NP or CGH alone and to those without neck pain. When more than one control group was available, data were preferentially extracted from the group most com-parable to the experimental sample in terms of sex distribution, occupational background, and other relevant characteristics.

### 2.3. Study Inclusion and Data Extraction

Two independent reviewers (PB, MP) screened all articles by title, abstract, and full text. Disagreements were resolved through consensus, and if necessary, consultation with a third reviewer (PB). For each eligible study, two reviewers (PB, MP) independently ex-tracted data, including author, year, country, sample size, participant demographics (age, sex), diagnostic criteria for cervical pain or cervicogenic headache, diagnostic criteria for TMDs, prevalence of TMDs in cases and controls, quantitative measures (e.g., pressure pain threshold), and main statistical outcomes. Authors of the included studies were contacted for data clarification when necessary.

Methodological quality was assessed using the Joanna Briggs Institute (JBI) Critical Appraisal Checklist for Analytical Cross-Sectional Studies [28], which evaluates sample selection, validity and reliability of measurements, control of confounding factors, and appropriateness of statistical analyses. Each item was rated as “Yes,” “No,” “Unclear,” or “Not applicable.” Two independent reviewers (PB, MP) performed the assessment, with disagreements resolved through discussion with a third reviewer.

### 2.4. Statistical Analysis and Meta-Analysis

Descriptive statistics were used to summarize study characteristics and TMD prevalence in participants with and without NP or CGH. Dichotomous outcomes were pooled as odds ratios using the Mantel–Haenszel method; computations were performed on the log(OR) scale for variance estimation and results were back-transformed to the natural OR scale for reporting, with two-sided 95% confidence intervals (α = 0.05). Continuous outcomes were pooled as mean differences when studies used identical scales, or as standardized mean differences (Hedges’ g, small-sample corrected) when scales differed. The primary model for all meta-analyzed outcomes was random-effects with DerSimonian–Laird estimation of the between-study variance (τ^2^). For continuous data, when only standard errors or 95% confidence intervals were reported [29], standard deviations were derived using standard formulas prior to pooling. Between-study heterogeneity was summarized using Cochran’s Q, I^2^, and τ^2^.

Where at least two studies reported comparable outcomes with sufficient data, a random-effects meta-analysis was planned to account for expected heterogeneity. Heterogeneity was quantified with the I^2^ and τ^2^ statistics. We interpreted between-study heterogeneity using the following a priori thresholds commonly applied in meta-analyses [30]: I^2^ = 0% (none), 0–25% (low), 25–50% (moderate), and >50% (high) [31]. Publication bias was assessed using funnel plots. Because each outcome involved fewer than 10 studies, formal asymmetry tests (e.g., Egger’s, Begg’s) were not performed due to low power and inflated false-positive risk [27]. Statistical analyses were performed using Review Manager 5.3 (RevMan, The Nordic Cochrane Centre, The Cochrane Collaboration, Copenhagen, Denmark). Certainty of evidence was assessed using the GRADE approach, starting observational evidence at low and rating down for risk of bias, inconsistency, indirectness, imprecision, and publication bias.

## 3. Results

**Figure 1:**
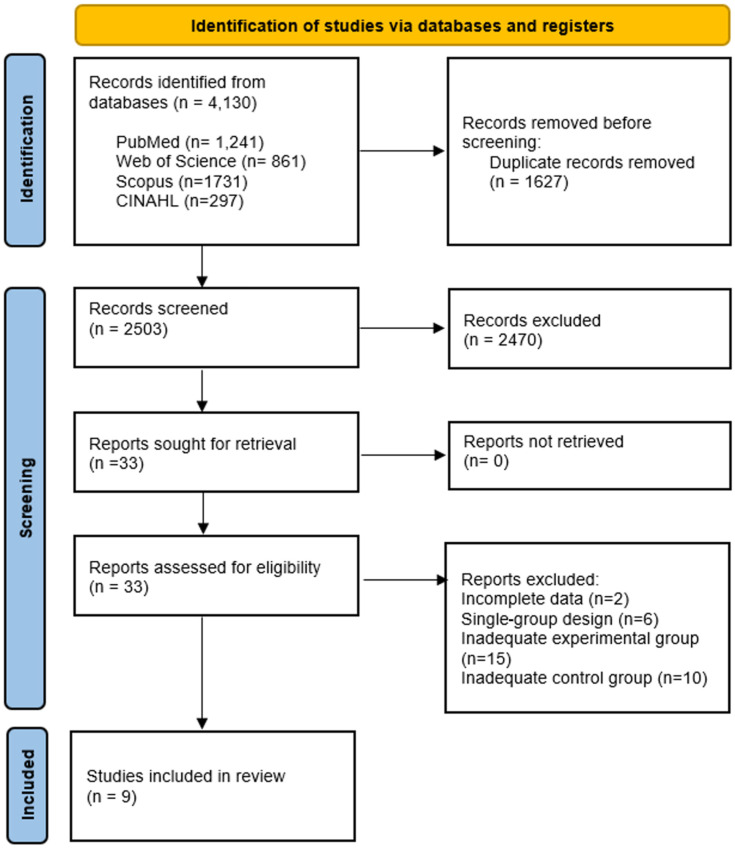
PRISMA flow-chart.

The initial search yielded 4130 records, which were reduced to 2503 after removal of duplicates. Following title and abstract screening, 33 articles were assessed in full text for eligibility. Six studies were excluded due to a single-arm design, fifteen because the experimental group did not meet eligibility criteria, and ten because of inadequate control groups. Two studies reported incomplete data. The corresponding authors were contacted for clarification, but we received no replies; therefore, these studies were excluded. Ultimately, nine studies fulfilled the inclusion criteria and were incorporated into this systematic review [29,32,33,34,35,36,37,38,39]. The study selection process is summarized in a PRISMA flow diagram (Figure 1).

Studies were conducted in Brazil [29,38,39], Spain [32,34,36,37], Turkey [33], and Belgium [35], with data collected in university communities, in physical therapy clinics, or via general population recruitment. Four studies [29,35,38,39] exclusively included female participants, while others reported female representation from 50% to 90% per group. NP was consistently defined by symptom duration (≥3 months), Neck Disability Index (NDI) scores (≥4), and pain intensity (NPRS/VAS ≥ 3). All articles regarding neck pain included chronic patients (symptom duration ≥ 3 months). In the study by Mingels [35] and colleagues, the diagnosis of CGH was established according to the ICHD-3 criteria [6]. Common exclusion criteria encompassed previous trauma, surgery, radiculopathy, fibromyalgia, pre-existing orofacial pain, medication use, and high psychological distress. Asymptomatic controls were defined by the absence of NP or headache. With the exception of the three studies assessing TMD prevalence [29,33,38], all studies reported the presence of TMDs or orofacial pain as exclusion criteria for both experimental and control groups. The study characteristics are reported in Table 1.

The main outcomes investigated were the prevalence of TMDs, orofacial PPTs, presence of myofascial trigger points, and mandibular mobility. Certainty of evidence is reported in Table 2.

### 3.1. TMD Prevalence

Three studies reported TMD prevalence in both experimental and control groups. All studies included participants with chronic neck pain in the experimental cohort and employed the Research Diagnostic Criteria for Temporomandibular Disorders (RDC/TMD) for diagnostic assessment. Two articles [29,38] exclusively enrolled female participants, while in the study by Guzel [33], females accounted for 51% of the control group and 58% of the CNP group.

**Figure 2:**
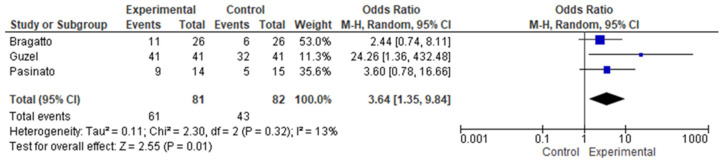
Meta-analysis of TMD prevalence. Data derived from the included studies [29,33,38]. Blue squares show study-specific odds ratios (size proportional to weight) with 95% confidence intervals. The black diamond shows the pooled random-effects estimate (95% CI), and the vertical line at OR = 1 indicates no effect.

Consistently across studies, the prevalence of TMDs was higher among participants with CNP relative to healthy controls. Meta-analysis of three studies demonstrated an increased risk of TMDs in individuals with CNP compared with controls, with low heterogeneity (pooled prevalence: 68.9% vs. 44.8%; OR = 3.64, 95% CI 1.35–9.84; I^2^ = 13%) (Figure 2). Subgroup analyses by specific TMD subtypes could not be conducted due to incomplete data reporting.

### 3.2. Jaw Mobility

**Figure 3:**
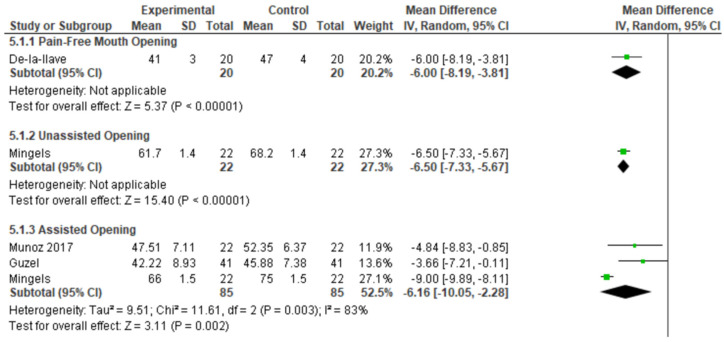
Meta-analysis of mouth opening. Data derived from the included studies [32,33,35,36]. Green squares indicate study-specific mean differences (size proportional to weight) with horizontal lines showing 95% CIs; black diamonds show the pooled random-effects estimates (width = 95% CI), and the vertical line at 0 indicates no effect.

Mandibular mobility was assessed in four studies [32,33,35,36], which reported reduced ranges of movement in patients with NP or CGH (Figure 3). All four studies reported results for jaw opening, measured in three studies using a ruler and in one with a caliper. One study assessed jaw opening in patients with CGH compared to controls. De-la-Llave-Rincón et al. (2012) [32] observed that pain-free opening was significantly smaller in the NP group (41 ± 3 mm) compared with controls (47 ± 4 mm, *p* < 0.01). In Mingels et al. (2019) [35], unassisted opening was 61.7 ± 1.4 mm in CGH patients versus 68.2 ± 1.4 mm in controls (*p* < 0.01). Meta-analysis of three studies reporting maximum assisted mouth (MMO) opening showed a mean difference of 6.16 mm (−10.05, −2.28) between study groups and controls, with high heterogeneity (I^2^ = 83%). Funnel plot of the meta-analysis on assisted mouth opening are reported in Supplementary Material (Figure S1). Guzel and colleagues [33] also assessed right and left laterotrusion and protrusion, reporting lower values in patients compared to asymptomatic controls (Table S1).

### 3.3. Masticatory Muscle PPTs

Pressure pain thresholds (PPTs) were consistently evaluated with pressure algometers to quantify mechanical hyperalgesia across different cranio-mandibular anatomical sites. Three studies [29,34,36] assessed PPTs over the masseter muscle and five studies [29,34,35,37,39] over the temporalis muscle. With the exception of the study by La Touche et al. [34], measurements were performed over specific muscle regions [29,35,37,39]. Both digital and mechanical algometers were employed, and PPT values were reported using different units, including grams per square centimeter (g/cm^2^) [29], kilograms per square centimeter (kg/cm^2^) [34,36,39], and kilopascals per square centimeter (kPa/cm^2^) [35]. Bragatto et al. [29] conducted bilateral PPT assessments but reported data only for the right side. The corresponding authors were approached for clarification, but additional data could not be obtained.

**Figure 4:**
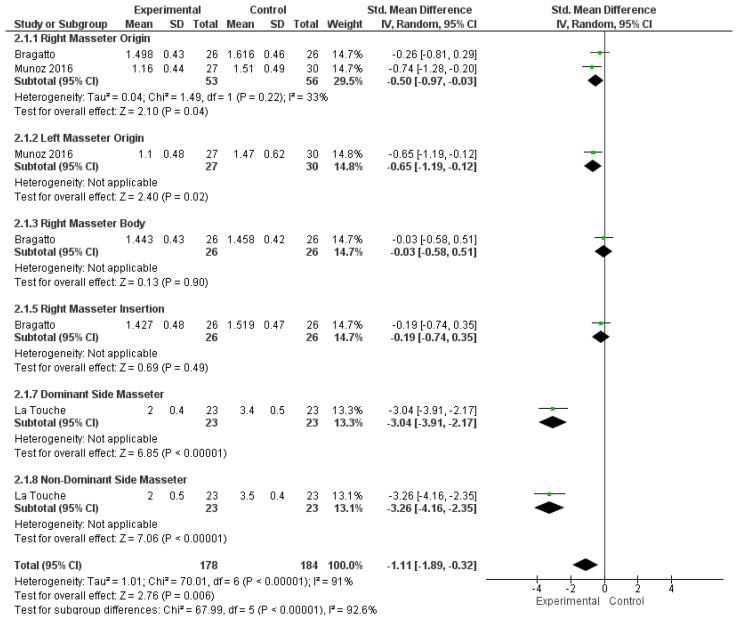
Meta-analysis of masseter pressure pain threshold. Data derived from the included studies [29,34,37]. Green squares indicate individual study standardized mean differences (size proportional to weight) with horizontal lines indicating 95% Cis. Black diamonds show the pooled random-effects estimates (width = 95% CI) for each subgroup and overall, and the vertical line at 0 indicates no effect.

**Figure 5:**
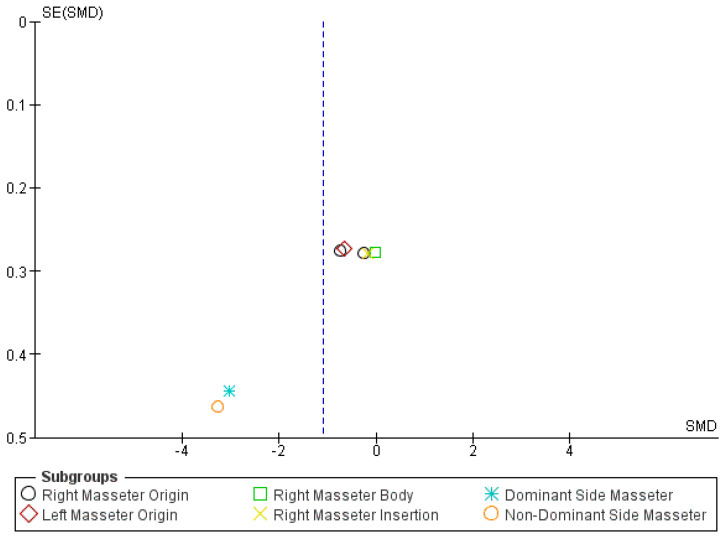
Funnel plot of meta-analysis of masseter pressure pain threshold. Dominant and non-dominant side masseter are derived from the included study of La Touche and colleagues [34]. The blue dashed vertical line indicates the pooled (overall) standardized mean difference.

Pooled analysis of masseter PPTs demonstrated increased mechanical sensitivity in individuals with neck pain compared to controls (SMD −1.11; 95% CI −1.89 to −0.32), corresponding to a large effect size with high heterogeneity (I^2^ = 92.6%) (Figure 4). After exclusion of the study by La Touche, based on potential publication bias identified through visual inspection of the funnel plot and forest plot (Figure 5), the pooled SMD for masseter PPTs de-creased to −0.38 (95% CI −0.65 to −0.11), indicating a moderate effect size with low heterogeneity (I^2^ = 8%).

**Figure 6:**
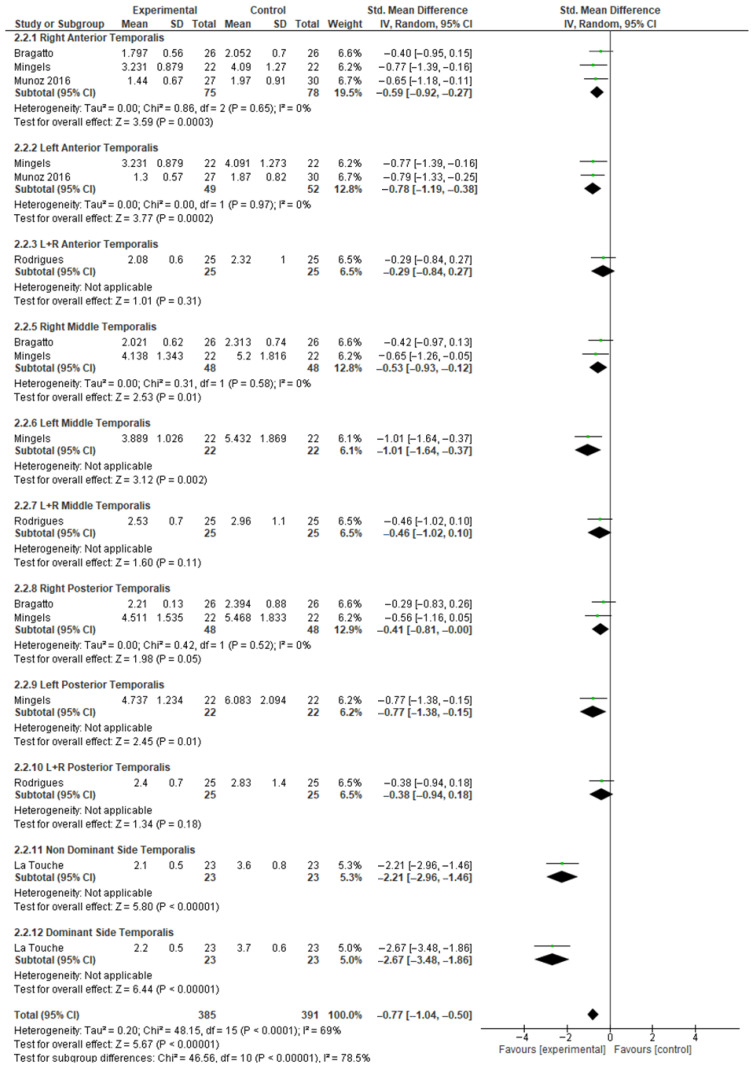
Meta-analysis of temporalis pressure pain threshold. Data derived from the included studies [29,34,35,37,39]. Green squares indicate individual study standardized mean differences (size proportional to weight) with horizontal lines indicating 95% Cis. Black diamonds show the pooled random-effects estimates (width = 95% CI) for each sub-group and overall, and the vertical line at 0 indicates no effect.

**Figure 7:**
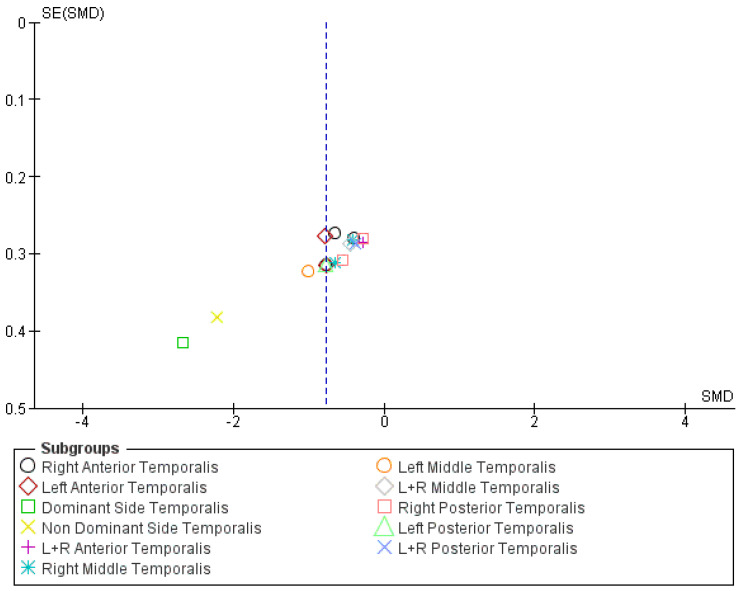
Funnel plot of meta-analysis of temporalis pressure pain threshold. Dominant and non-dominant side temporalis are derived from the included study of La Touche and colleagues [34]. The blue dashed vertical line indicates the pooled (overall) standardized mean difference.

Pooled analysis of temporalis PPTs indicated increased mechanical sensitivity in individuals with neck pain or CGH compared with controls (SMD −0.77; 95% CI −1.04 to −0.50) (Figure 6), reflecting a moderate effect size with high heterogeneity (I^2^ = 69%). After exclusion of the study by La Touche et al. (Figure 7), the pooled SMD for temporalis PPTs was −0.57 (95% CI −0.72 to −0.42), corresponding to a moderate effect size with no heterogeneity (I^2^ = 0%).

### 3.4. Pain at Palpation and Myofascial Pain

The anatomical sites assessed for pain at palpation included a wide range of craniofacial structures. Methods of assessment varied across studies: some studies applied intensity scales such as the Numerical Pain Rating Scale (NPRS) [29] or a binary response (“pain” vs. “no pain”) [33,35]. For the diagnosis of myofascial trigger points (TrPs), authors used the procedure described by Gerwin et al. [32,40].

De-la-Llave-Rincón et al. (2012) [32] reported a significantly greater number of latent TrPs in the masticatory muscles (masseter and temporalis) of patients with CNP compared with controls (median four TrPs vs. one TrP, *p* < 0.001). Similarly, Guzel [33] also found higher palpation pain responses in orofacial regions of patients compared with healthy controls. Complete data are presented in Table S2—Supplementary Material.

### 3.5. Methodological Quality

The methodological quality of the included cross-sectional studies was assessed using the JBI Critical Appraisal Checklist (Table 3). All studies fulfilled minimum quality standards and were retained in this review, although limitations were noted. Inclusion criteria, populations, and settings were generally well defined, and validated instruments were consistently employed for outcome assessment. The main methodological weakness concerned the handling of confounders, which were often not identified or adequately controlled. Only a few studies, such as La Touche et al. (2010) [34] and Rodrigues et al. (2024) [39], incorporated covariates or stratified designs. Overall, the studies were considered methodologically acceptable, but their constraints should be considered when interpreting the findings.

## 4. Discussion

This systematic review synthesized current evidence on the prevalence and clinical features of temporomandibular disorders, mandibular ROM, orofacial pressure pain thresholds (PPTs), and orofacial pain at palpation in individuals with NP and/or CGH compared with asymptomatic controls. Across studies, patients with cervical pain or CGH consistently exhibited higher TMD prevalence, reduced PPTs, increased frequency of temporomandibular trigger points, and decreased mandibular mobility, supporting a significant clinical association likely underpinned by shared neurophysiological mechanisms, with potential biomechanical consequences [34].

Our results are aligned with evidence from headache disorders, where migraine and tension-type headache are associated with higher risk of painful or mixed TMDs, and vice versa [11,41]. Collectively, these findings highlight the clinical relevance of comprehensive assessment strategies addressing both craniofacial and cervical regions in patients with craniocervical symptoms.

The pathophysiological link between these conditions arises from convergence of trigeminal and upper cervical (C1–C3) nociceptive afferents within the trigeminocervical nucleus. This integration promotes central sensitization and regional symptoms [12]. The trigeminocervical complex (TCC) constitutes the substrate where trigeminal and cervical inputs meet, facilitating cross-sensitization and reciprocal pain referral, thus explaining symptom overlap and diagnostic challenges [42].

CGH should not be viewed as separate from cervical musculoskeletal pain, since both share the same pathophysiological basis in the TCC. Afferents from C2 and C3 converge onto second-order neurons, enabling referral of cervical nociception as headache and fostering sensitization [43]. This mechanism explains how cervical disorders may manifest as CGH and aligns with ICHD-3 criteria attributing headache to neck pathology, supporting a continuum rather than distinct entities [44,45].

Several studies explicitly excluded diagnosed TMD to isolate the effects of NP or CGH on orofacial outcomes [32,34,35,36,37,39]. On one hand, this approach strengthens internal validity for hypotheses such as trigeminal sensitization. On the other hand, it reduces external generalizability, since TMD and NP frequently coexist. Therefore, these studies may even underestimate the true prevalence and severity of TMD manifestations in general clinical populations, and their clinical impact.

Furthermore, we observed consistently reduced orofacial PPTs in the experimental groups, reflecting heightened pain sensitivity in the masticatory muscles and adjacent structures. This is a typical manifestation of peripheral and central sensitization [12], where receptive fields expand beyond territories as a consequence of altered neuronal properties within spinal nociceptive pathways. Importantly, sensitization processes appear to develop even without self-reported orofacial pain, suggesting that underlying neurophysiological alterations can occur in the absence of a defined pain diagnosis. From a clinical point of view, PPTs over the masseter showed significant negative correlations with both NP intensity and duration [34], emphasizing its relevance in both evaluation and consequently management of NP or CGH patients.

Even if orofacial influence in NP should be taken into account, it appears to play the role of a contributing factor in cervical disorders more than being the primary source of neck pain. Reductions in PPTs compared to controls were observed to be greater at the cervical level (C5–C6 joint) compared with trigeminal sites, reflecting greater cervical sensitization [34].

Mandibular musculoskeletal impairments, with reduced maximal mouth opening and restricted excursions, were associated with NP or CGH in this review. These findings reinforce the hypothesized association between neck pain and subclinical jaw impairments, which may occur even in the absence of specific TMD diagnoses or orofacial pain. Moreover, the literature suggests that musculoskeletal impairments of the jaw and the neck may be linked in neck pain populations. Reduced cervical muscle strength and endurance and reduced range of movement correlated with pain severity in the studies included in this review [33,38]. Such findings may be explained by reflex inhibition associated with pain and altered motor control strategies. Central sensitization has direct motor consequences, and the excitement of trigeminal motoneurons can result in involuntary synergistic co-contractions of the masticatory muscles, thereby restricting movement. This hypothesis is supported by a significant negative association between headache frequency and maximal mouth opening in subjects with CGH [35], and pain duration and intensity in NP subjects [34].

Moreover, the burden of NP seems to be influenced by the coexistence of TMDs and NP, with higher Neck Disability Index (NDI) scores [29]. Conversely, TMD-directed therapies, including TMJ injection, physiotherapy, and musculoskeletal interventions, have been observed to improve headache intensity, neck pain, and neck disability in patients with orofacial and cervical comorbidities [23,46,47].

These painful comorbidities are not limited to peripheral dysfunction but may also reflect amplified central sensitization, as indicated by widespread hyperalgesia and a higher number of pain sites. Patients with concurrent TMDs and NP frequently report diffuse pain distribution. In line with these findings, Muñoz-García et al. (2017) [36] demonstrated that individuals with TMDs and CNP presented with a greater number of widespread pain sites compared to those with CNP alone, suggesting impaired central pain modulation. The widespread distribution of this hypersensitivity, also affecting extra-trigeminal sites such as the anterior tibialis muscle area [35,37], emphasizes that these alterations may involve supraspinal pain mechanisms, possibly being included in a generalized painful condition typical in central sensitization.

Beyond musculoskeletal impairments and symptoms, psychosocial and behavioral factors may play a crucial role in the interaction between TMDs and cervical pain, and explain part of a generalized painful clinical profile, with impaired descending pain modulation. Patients with concurrent TMDs and neck pain consistently report higher levels of pain catastrophizing, anxiety, and perceived stress [36,37]. These factors are well-established contributors to pain chronification, promoting maladaptive coping strategies and impairment [48]. Within the DC/TMD classification [1], psychosocial assessment is included as Axis II, highlighting the recognized impact of psychosocial factors on temporomandibular disorders [49]. Nevertheless, the detailed evaluation of these characteristics extends beyond the specific objectives of the present systematic review.

Although our search did not yield studies assessing the prevalence of bruxism among individuals with NP or CGH, bruxism has been proposed as a possible mechanism linking cervical and temporomandibular dysfunction [50]. Awake bruxism [51], through increased masticatory muscle activation and joint loading, may exacerbate cervical impairments by reinforcing recruitment of cervical stabilizers and perpetuating muscle pain [20]. Elevated pain responses during palpation of masticatory muscles and nociception originating in neck muscles may also be explained by myogenic overload, including excessive or prolonged loading in individuals with chronic neck pain [52]. Although not assessed across the included studies, bruxism represents a potentially modifiable behavioral factor contributing to pain persistence in the upper quadrant region [53].

### 4.1. Clinical Implications

The findings highlight the necessity of a multidisciplinary approach for patients presenting with cervical pain, CGH, or TMD. Clinical assessment should extend beyond regional examination to include palpation of masticatory and cervical muscles, evaluation of mandibular and cervical mobility, and screening for psychosocial distress and parafunctional behaviors. Given the evidence of neuroanatomical convergence and central sensitization [12], therapeutic strategies targeting one system are likely to influence the other, as shown in other musculoskeletal disorders [54,55]. Integrated interventions, including manual therapy [56,57], exercise therapy [58,59], cognitive–behavioral support [53], and education on pain neuroscience and parafunctional habits [60], should be considered.

The findings underscore the need for integrated clinical management. Dentists, physiotherapists, neurologists, and other clinicians should routinely screen for TMD signs and symptoms in patients with cervical pain/CGH.

### 4.2. Strengths and Limitations

Several limitations must be considered when interpreting the results. Small sample sizes and reliance on convenience sampling, often involving predominantly young women [34,36], limit the generalizability of the findings. In some studies, exclusively female participants were recruited. Due to the higher prevalence of TMDs and orofacial symptoms in female populations, such findings restrict external validity to male populations.

Most studies applied unadjusted analyses for confounders, while effect sizes and power calculations were poorly reported. These limitations reduce the strength of the evidence. Moreover, the cross-sectional design of all included studies precludes causal inference and prevents the establishment of clear temporal or directional relationships between cervical pain/CGH and TMDs.

Finally, psychosocial and behavioral dimensions were not consistently assessed [61]. Key factors such as stress, anxiety, depression, and bruxism were seldom incorporated into study protocols. The limited integration of these variables is a critical gap, considering their established role in the biopsychosocial model of chronic pain.

This review synthesized evidence from nine analytical cross-sectional studies. Most of these investigations employed validated diagnostic criteria and outcome measures, thereby strengthening the reliability of the findings. Several studies also applied standardized protocols for the assessment of mandibular mobility, which enhanced methodological consistency across cohorts.

### 4.3. Future Research

Future research should prioritize prospective and longitudinal designs with larger, methodologically robust samples to establish causal relationships and clarify the natural progression of comorbid TMDs and cervical pain/CGH. Diverse cohorts should be included, encompassing both sexes and multiple TMD subtypes, to improve generalizability beyond the predominantly young female populations of existing studies.

Uniform use of validated diagnostic criteria is essential to enhance comparability across studies. Standardized frameworks such as the DC/TMD for temporomandibular disorders [1] and the ICHD-3 for headache classification [6] should be systematically applied to ensure methodological consistency and reliability of findings.

The contribution of bruxism [62], particularly awake bruxism, requires systematic investigation through validated and standardized assessment tools [50]. Clarifying its role as a mediator or comorbidity in the interaction between cervical and temporomandibular dysfunctions would provide important insights into both pathophysiology and management.

## 5. Conclusions

This review highlights an association between TMDs and orofacial symptoms, and NP or CGH. Methodological heterogeneity and the limitations of cross-sectional designs constrain causal inference. Future research should adopt longitudinal approaches and systematically integrate psychosocial dimensions to advance mechanistic understanding and optimize multimodal care.

## Figures and Tables

**Table 1:** Study characteristics: NP: neck pain; CNP: chronic neck pain; TMD: temporomandibular disorder; CI: confidence interval; NDI: Neck Disability Index; NPRS: Numeric Pain Rating Scale; GP: general practitioner; PT: physical therapy; VAS: Visual Analogic Scale; MSK: musculoskeletal; CGH: cervicogenic headache; TMJ: temporomandibular joint.

First Author (Year)	Country	Data Collection Setting	Sample Size and Mean Age per Group	Female Subjects per Group (%)	Inclusion Criteria	Exclusion Criteria
Bragatto (2016) [29]	Brazil	University Campus	NP: *n* = 26, mean 36.5 (CI 33.4–36.6) Controls: *n* = 26, mean 33.8 (CI 30.7–37.0)	100%	NP: Age 20–50 Same job ≥ 12 months Computer ≥ 4 h/day Self-reported neck pain (MUEQ-Br) Duration ≥ 3 months NDI ≥ 5 NPRS ≥ 3	Other disorders Systemic degenerative diseases, fibromyalgia Whiplash/trauma Congenital deformities Spinal surgery Leg discrepancy > 2 cm Uncorrected vision/hearing loss Fixed orthodontics Cognitive impairment Pregnancy/lactation/postpartum ≤ 1 yr Previous TMD
De-la-Llave-Rincon (2012) [32]	Spain	Physical Therapy Clinic	NP: n = 20, mean 27 ± 6 Controls: *n* = 20, mean 27 ± 5	NP: 60% Controls: 60%	NP: Insidious mechanical NP confirmed by GP Neck/shoulder pain provoked by posture/movement/palpation Bilateral ≥ 6 months Age 20–37 Controls: Asymptomatic volunteers, matched 20–37 y	Unilateral NP Orofacial pain Primary headache Fibromyalgia Whiplash Cervical surgery Radiculopathy/myelopathy Previous cervical PT Age < 18 Controls: history of NP/head/face pain, systemic disease, trauma Patients: analgesics/muscle relaxants ≤ 72 h; NPRS ≤ 3 days from exam
Guzel (2022) [33]	Turkey	Physical Therapy Clinic	CNP: *n* = 41, mean 33.6 ± 8.8 Controls: *n* = 41, mean 29.7 ± 5.8	CNP: 58% Controls: 51%	CNP: Age 20–50 NP ≥ 3 months VAS > 0 NDI ≥ 5 Controls: Age 20–50 No NP	MSK pain outside neck Cervical/TMD surgery Other cervical MSK disorders Specific pathology (TMJ malignancy, fracture, systemic rheumatic) Facial paralysis Active therapy for NP/TMD Psychiatric disease Communication difficulties
La Touche (2010) [34]	Spain	Physical Therapy Clinic	CNP: *n* = 23, mean 28 ± 5 Controls: *n* = 23, mean 28 ± 6	NP: 56% Controls: 56%	NP: Insidious mechanical NP confirmed by GP Neck/shoulder pain provoked by posture/movement/palpation Bilateral ≥ 6 months Age 20–37 Controls: Asymptomatic, matched age/sex Episodic infrequent TTH allowed	Unilateral NP Fibromyalgia Whiplash Cervical surgery Radiculopathy/myelopathy Previous cervical PT Severe arthritis (>30 y radiograph) Age < 18 TMD Primary headache Controls: NP/head/face pain, systemic disease, trauma (i.e., whiplash) Preventive meds/analgesics ≤ 72 h
Mingels (2019) [35]	Belgium	Recruitment by Public Call	CGH: *n* = 22, mean 20.7 ± 2.5 Controls: *n* = 22, mean 21 ± 2.3	100% women in all groups	CGH: Women, age 18–30 ICHD-3 CGH No TMD or headache attributed to TMD (ICHD-3, TMD Pain Screener) Cervical dysfunctions confirmed by neurologist Controls: Women 18–30, asymptomatic No TMD (TMD Pain Screener) Matched age and socio-economic status	Pregnancy PT for head/neck within 4 w Severe MSK/neuro/endocrine/cardiac/psychiatric Arm pain radiation Drug abuse Trauma to head/neck Orthodontics CGH: radiculitis/radiculopathy Controls: headache CGH VAS < 3 on exam day
Muñoz-García (2016) [37]	Spain	University Campus and Community	NP: *n* = 22, mean 25.6 ± 4.2 Controls: *n* = 22, mean 24 ± 4.6	NP: 50% Controls: 54.5%	NP: NPRS ≥ 3 last 3 m Isolated cervical pain, mechanical NP (provoked by posture/movement/palpation) Pain recognition procedures NDI ≥ 5 Age 18–65 Controls: Asymptomatic volunteers Age 18–65	Previous TMD surgery Rheumatic disease Severe TMJ pathology Neuropathic/odontogenic pain Psychosis Antidepressants/anxiolytics Narcotics Pregnancy Controls: NP/head/face pain, systemic disease
Muñoz-García (2017) [36]	Spain	Local Community	CNP: *n* = 27, mean 27.5 ± 4.8 Controls: *n* = 30, mean 26.2 ± 4.5	CNP: 63% Controls: 57%	CNP: Age 18–40 NP ≥ 12 w NP provoked by posture/movement/palpation Recognition of spinal pain NDI ≥ 5 NPRS ≥ 3 Controls: Age 18–40 No NP/face/head pain	Previous TMD surgery Rheumatism/whiplash Severe TMJ pathology Neuropathic/odontogenic pain Psychosis Antidepressants/anxiolytics Narcotics Pregnancy Controls: med use, systemic disease Caffeine/alcohol ≤ 48 h
Pasinato (2016) [38]	Brazil	Local Community	NP: *n* = 14, mean 27.5 ± 3.9 Controls: *n* = 15, mean 25.4 ± 6.0	100% women	NP: NP > 3 months NDI > 4 Age 21–42 Controls: No NP NDI ≤ 4 Age 21–42	Trauma/fracture/surgery in region Radiculopathy/myelopathy Fibromyalgia, inflammatory arthritis Strengthening exercise for neck/upper limb ≤ 6 m
Rodrigues (2024) [39]	Brazil	Faculty of Medicine	Controls: *n* = 25, mean 28.5 ± 7.2 CNP: *n* = 25, mean 32.9 ± 10.2	100% women	CNP: Women aged 18–55 Chronic NP ≥ 3 m without arm pain NDI ≥ 4 NPRS ≥ 4 No headache last year Controls: Women aged 18–55 No NP/headache last year	Men Persistent NP/headache Other primary/secondary headaches Analgesic abuse Cervical/facial trauma Pregnancy Cervical disc disease Systemic disease Anesthetic blocks ≤ 3 m

**Table 2:** Summary of findings (GRADE). a = risk of bias: confounding not adequately controlled; b = imprecision: wide CI and PI; c = inconsistency: moderate-to-high heterogeneity and PI crossing the null. A complete summary of the findings is reported in the Supplementary Material (Table S2). TMD: temporomandibular disorder; NP: neck pain; MD: mean difference; SMD: standa rdized mean difference; CI: confidence interval; OR: odds ratio; GRADE certainty: ⬤⬤⬤⬤ high, ⬤⬤⬤◯ moderate, ⬤⬤◯◯ low, ⬤◯◯◯ very low.”

Outcome	Population/Comparator	K	Effect (95% CI)	Heterogeneity (I^2^/τ^2^)	95% Prediction Interval	Certainty (GRADE)	Footnotes
TMD prevalence	Adults with chronic NP vs. asymptomatic controls	k = 3	OR 3.64 (1.35–9.84)	I^2^ 13%/τ^2^ 0.11	0.27–49.29	Very Low ⬤◯◯◯	a, b, c
Mouth opening (mm)	Adults with chronic NP/CGH vs. asymptomatic controls	k = 5	MD −6.16 mm (−10.05 to −2.28)	I^2^ 84%/τ^2^ 2.88	−14.46 to 2.14	Very Low ⬤◯◯◯	a, b, c
Masseter pressure pain threshold	Adults with chronic NP vs. asymptomatic controls	k = 7	SMD −1.11 (−1.89 to −0.32)	I^2^ 91%/τ^2^ 1.01	−3.89 to 1.67	Very Low ⬤◯◯◯	a, b, c
Temporalis pressure pain threshold	Adults with chronic NP/CGH vs. asymptomatic controls	k = 16	SMD −0.77 (−1.04 to −0.50)	I^2^ 69%/τ^2^ 0.20	−1.77 to 0.23	Very Low ⬤◯◯◯	a, b, c

**Table 3:** Methodological quality assessment of the included studies according to the JBI critical appraisal checklist.

Study	Q1: Inclusion Criteria clearly Defined?	Q2: Study Subjects and Setting Described in Detail?	Q3: Exposure Measured Validly and Reliably?	Q4: Objective, Standard Criteria for Condition?	Q5: Confounders Identified?	Q6: Strategies to Deal with Confounders Stated?	Q7: Outcomes Measured Validly and Reliably?	Q8: Appropriate Statistical Analysis Used?
Bragatto et al., 2016 [29]	Yes	Yes	Yes	Yes	Unclear	Unclear	Yes	Unclear
De-la-Llave-Rincón et al., 2012 [32]	Yes	Yes	Yes	Yes	Unclear	Unclear	Yes	Unclear
Güzel et al., 2022 [33]	Yes	Yes	Yes	Yes	Unclear	Unclear	Yes	Unclear
La Touche et al., 2010 [34]	Yes	Yes	Yes	Yes	Yes	Yes	Yes	Yes
Mingels et al., 2019 [35]	Yes	Yes	Yes	Yes	Yes	Unclear	Yes	Yes
Muñoz-García et al., 2016 [37]	Yes	Yes	Yes	Yes	Unclear	Unclear	Yes	Unclear
Muñoz-García et al., 2017 [36]	Yes	Yes	Yes	Yes	Unclear	Unclear	Yes	Unclear
Pasinato et al., 2016 [38]	Yes	Yes	Yes	Yes	Unclear	Unclear	Yes	Unclear
Rodrigues et al., 2024 [39]	Yes	Yes	Yes	Yes	Yes	Unclear	Yes	Yes

## Data Availability

No additional datasets were generated for this manuscript.

## Supplementary Materials

The following supporting information can be downloaded at https://www.mdpi.com/article/10.3390/jcm15010266/s1, Figure S1: Funnel plot of the meta-analysis on assisted mouth opening; Table. S1: Study results; Table S2: Summary of Findings (GRADE).; Table S3: Search Strategy; Table S4: PRISMA checklist.

## Abbreviations

The following abbreviations are used in this manuscript:

TMD	Temporomandibular Disorder
NP	Neck Pain
CNP	Chronic Neck Pain
CGH	Cervicogenic Headache
TMJ	Temporomandibular Joint
IHS	International Headache Society
RDC/TMD	Research Diagnostic Criteria for Temporomandibular Disorders
DC/TMD	Diagnostic Criteria for Temporomandibular Disorders
OR	Odds Ratio
PPT	Pressure Pain Threshold
CI	Confidence Interval
NDI	Neck Disability Index
NPRS	Numeric Pain Rating Scale
GP	General Practitioner
PT	Physical Therapy
VAS	Visual Analogic Scale
SMD	Standardized Mean Difference
