# Supplementary material for: Temporomandibular Disorders and Orofacial Outcomes in Subjects with Neck Pain and/or Cervicogenic Headache: A Systematic Review with Meta-Analysis
Source: J Clin Med. 2025 Dec 29;15(1):266. doi: 10.3390/jcm15010266 (PMC12786625; doi:10.3390/jcm15010266)
Supplement: Supplementary file 1 [file jcm-15-00266-s001.zip › jcm-3917931-supplementary 2nd proofreading_PB.pdf]

Table S1 – Study results; RDC/TMD – Research Diagnostic Criteria for Temporomandibular Disorders; PPT: Pressure Pain Threshold; * p<0.05; ** p<0.01; *** p<0.001; NS: Non-significant differences between experimental and control groups

Study	TMD Prevalence	Jaw Mobility	Masseter muscle PPT	Temporalis Muscle PPT	Pain at Palpation	TMJ Noises
Bragatto et al., 2016 [29]	RDC/TMD	-	Right Masseter<br><br>Origin **NS**<br>Body **NS**<br>Insertion **NS**	Right Temporalis<br><br>Anterior **NS**<br>Middle **NS**<br>Posterior **NS**	NPRS (Bilateral):<br><br>Anterior temporalis muscle **NS**<br>Middle temporalis muscle **NS**<br>Posterior temporalis muscle **NS**<br>Masseter muscle origin **NS**<br>Masseter muscle belly **NS**<br>Masseter muscle insertion **NS**<br>Submandibular region (suprahyoids) **NS**<br>Posterior mandible site **NS**<br>Temporalis muscle tendon **NS**<br>TMJ lateral pole **NS**<br>TMJ posterior pole **NS**	-
De-la-Llave-Rincón et al., 2012 [32]	-	Pain Free Mouth Opening***	-	-	Myofascial Trigger Points:<br><br>Masseter***<br>Temporalis***	-
Güzel et al., 2022 [33]	RDC/TMD	Maximum assisted mouth opening *<br>Maximum right laterotrusion***<br>Maximum left laterotrusion**<br>Maximum Protrusion**	-	-	Pain Yes/No<br><br>Right Masseter **NS**<br>Left Masseter**<br>Right Temporalis*<br>Left Temporalis*<br>Right Lateral Pterygoid**<br>Left Lateral Pterygoid***<br>Right Medial Pterygoid***<br>Left Medial Pterygoid***<br>TMJ***	Clicking Sound**<br>Crepitus **NS**
La Touche et al., 2010 [34]	-	-	Dominant Side***<br>Non Dominant Side***	Dominant Side Temporalis***<br>Non Dominant Side Temporalis***	-	-
Mingels et al., 2019 [35]	-	Unassisted Mouth Opening**<br><br>Maximum assisted mouth opening **	-	Right Temporalis<br>Anterior*<br>Middle*<br>Posterior*<br><br>Left Temporalis<br>Anterior**<br>Middle*<br>Posterior*	Pain Yes/No<br><br>Origin Right Masseter**<br>Body Right Masseter*<br>Insertion Right Masseter **NS**<br>Origin Left Masseter**<br>Body Left Masseter**<br>Insertion Left Masseter***<br><br>TMJ<br>Left **NS**<br>Right **NS**	-
Muñoz-García et al., 2017 [36]	-	Maximum assisted mouth opening**	-	-	-	-
Muñoz-García et al., 2016 [37]	-	-	Origin Right Masseter*<br>Origin Left Masseter*	Right Anterior*<br>Left Anterior**	-	-
Pasinato et al., 2016 [38]	RDC/TMD	-	-	-	-	-
Rodrigues et al., 2024 [39]	-	-	-	L+R Temporalis<br>Anterior **NS**<br>Middle **NS**<br>Posterior **NS**	-	-

Table S2. Summary of Findings (GRADE). a = Risk of bias: confounding not adequately controlled; b = Imprecision: wide CI and PI; c = Inconsistency: moderate-to-high heterogeneity and PI crossing the null; d = Imprecision: few small studies. Complete summary of findings is reported in Supplementary material. TMD: Temporomandibular disorders; NP: Neck Pain; MD: Mean Difference; SMD: Standardized Mean Difference; CI: Confidence Interval; OR: Odds Ratio

Outcome	Population/Comparator	K	Effect (95% CI)	Heterogeneity (I^2 / τ^2)	95% Prediction Interval	Certainty (GRADE)	Footnotes
TMD prevalence	Adults with chronic NP vs asymptomatic controls	k = 3	OR 3.64 (1.35–9.84)	I^2 13% / τ^2 0.11	0.27–49.29	Very Low ●○○○	a,b,c
Mouth opening (mm)	Adults with chronic NP/CGH vs asymptomatic controls	k = 5	MD −6.16 mm (−10.05 to −2.28)	I^2 84% / τ^2 2.88	−14.46 to 2.14	Very Low ●○○○	a,b,c
Masseter pressure-pain threshold	Adults with chronic NP vs asymptomatic controls	k = 7	SMD −1.11 (−1.89 to −0.32)	I^2 91% / τ^2 1.01	−3.89 to 1.67	Very Low ●○○○	a,b,c
Temporalis pressure-pain threshold	Adults with chronic NP/CGH vs asymptomatic controls	k = 16	SMD −0.77 (−1.04 to −0.50)	I^2 69% / τ^2 0.20	−1.77 to 0.23	Very Low ●○○○	a,b,c
Pain at palpation (TMJ/masticatory muscles)	Adults with chronic NP vs asymptomatic controls	k = 3	Higher frequency in cases	—	n/a	Very Low ●○○○	a,d
Myofascial trigger points	Adults with chronic NP vs asymptomatic controls	k = 1	Higher number of latent TrPs	—	n/a	Very Low ●○○○	a,d
Laterotrusion	Adults with chronic NP vs asymptomatic controls	k = 1	Reduced vs controls	—	n/a	Very Low ●○○○	a,d
Protrusion	Adults with chronic NP vs asymptomatic controls	k = 1	Reduced vs controls	—	n/a	Very Low ●○○○	a,d

Table S3 - Search Strategy

DATABASE	STRINGA
**PubMed**	("Neck Pain"[MeSH] OR "Cervical Pain" or "cervicogenic headache" or (cervical and headache)) and (("FACIAL PAIN"[All Fields] OR "FACIAL PAIN"[MeSH Terms] OR "temporomandibular joint dysfunction syndrome"[MeSH Terms] OR "craniomandibular disorders"[MeSH Terms] OR "temporomandibular joint"[MeSH Terms] OR "temporomandibular joint disorders"[MeSH Terms] OR "craniomandibul*"[All Fields] OR "temporomandibul*"[All Fields] OR "Temporomandibular pain"[All Fields] OR "Jaw dysfunction"[All Fields] OR "Jaw pain"[All Fields] OR "craniomandibular dysfunction*"[All Fields] OR "Craniomandibular pain"[All Fields] OR "Orofacial Pain"[All Fields] OR "TMJ"[All Fields] OR "TMD"[All Fields] OR "Temporomandibular Dysfunction"[All Fields] OR "Temporomandibular Joint Dysfunction"[All Fields] OR "Temporomandibular joint syndrome"[All Fields] OR "temporomandibular joint disorder*"[All Fields] OR "temporomandibular disorder*"[All Fields] or bruxism[mesh]) or ((Jaw or temporomandibular or mandibular or trigeminal or masseter or temporalis) and ("Pain Threshold"[Mesh] or "Range of Motion, Articular"[Mesh] OR "range of movement" or "Myofascial Pain Syndromes"[Mesh] or "Trigger Points"[Mesh] or Proprioception or sensitization or "motor control" or "mouth opening" or protrusion or laterotrusion)))
**EMBASE**	('Neck Pain'/exp OR 'Cervical Pain' OR 'cervicogenic headache') AND (('FACIAL PAIN' OR 'FACIAL PAIN'/exp OR 'temporomandibular joint dysfunction syndrome'/exp OR 'craniomandibular disorders'/exp OR 'temporomandibular joint'/exp OR 'temporomandibular joint disorders'/exp OR craniomandibul* OR temporomandibul* OR 'Temporomandibular pain' OR 'Jaw dysfunction' OR 'Jaw pain' OR 'craniomandibular dysfunction*' OR 'Craniomandibular pain' OR 'Orofacial Pain' OR TMJ OR TMD OR 'Temporomandibular Dysfunction' OR 'Temporomandibular Joint Dysfunction' OR 'Temporomandibular joint syndrome' OR 'temporomandibular joint disorder*' OR 'temporomandibular

DATABASE	STRINGA
	disorder*' OR bruxism/exp) OR ((Jaw OR temporomandibular OR mandibular OR trigeminal OR masseter OR temporalis) AND ('Pain Threshold'/exp OR 'Range of Motion, Articular'/exp OR 'range of movement' OR 'Myofascial Pain Syndromes'/exp OR 'Trigger Points'/exp OR Proprioception OR sensitization OR 'motor control' OR 'mouth opening' OR protrusion OR laterotrusion)))
**Scopus**	("Neck Pain" OR "Cervical Pain" OR "cervicogenic headache") AND (("FACIAL PAIN" OR "temporomandibular joint dysfunction syndrome" OR "craniomandibular disorders" OR "temporomandibular joint" OR "temporomandibular joint disorders" OR craniomandibul OR temporomandibul OR "Temporomandibular pain" OR "Jaw dysfunction" OR "Jaw pain" OR "craniomandibular dysfunction" OR "Craniomandibular pain" OR "Orofacial Pain" OR TMJ OR TMD OR "Temporomandibular Dysfunction" OR "Temporomandibular Joint Dysfunction" OR "Temporomandibular joint syndrome" OR "temporomandibular joint disorder" OR "temporomandibular disorder" OR bruxism) OR ((Jaw OR temporomandibular OR mandibular OR trigeminal OR masseter OR temporalis) AND ("Pain Threshold" OR "Range of Motion, Articular" OR "range of movement" OR "Myofascial Pain Syndromes" OR "Trigger Points" OR Proprioception OR sensitization OR "motor control" OR "mouth opening" OR protrusion OR laterotrusion)))
**CINAHL**	("Neck Pain" OR "Cervical Pain" OR "cervicogenic headache") AND (("temporomandibular disorders" OR "Temporomandibular pain" OR "Orofacial Pain" OR "temporomandibular disorder" OR bruxism OR ((Jaw OR temporomandibular OR mandibular OR trigeminal OR masseter OR temporalis) AND ("Pain Threshold" OR "Range of Motion, Articular" OR "range of movement" OR "Myofascial Pain Syndromes" OR "Trigger Points" OR Proprioception OR sensitization OR "motor control" OR "mouth opening" OR protrusion OR laterotrusion)))

Table S4 – PRISMA checklist

Section and Topic	Item #	Checklist item	Location where item is reported
**TITLE**			
Title	1	Identify the report as a systematic review.	1
**ABSTRACT**			
Abstract	2	See the PRISMA 2020 for Abstracts checklist.	1
**INTRODUCTION**			
Rationale	3	Describe the rationale for the review in the context of existing knowledge.	2
Objectives	4	Provide an explicit statement of the objective(s) or question(s) the review addresses.	3
**METHODS**			
Eligibility criteria	5	Specify the inclusion and exclusion criteria for the review and how studies were grouped for the syntheses.	4
Information sources	6	Specify all databases, registers, websites, organisations, reference lists and other sources searched or consulted to identify studies. Specify the date when each source was last searched or consulted.	3
Search strategy	7	Present the full search strategies for all databases, registers and websites, including any filters and limits used.	Supplementary material
Selection process	8	Specify the methods used to decide whether a study met the inclusion criteria of the review, including how many reviewers screened each record and each report retrieved, whether they worked independently, and if applicable, details of automation tools used in the process.	4
Data collection process	9	Specify the methods used to collect data from reports, including how many reviewers collected data from each report, whether they worked independently, any processes for obtaining or confirming data from study investigators, and if applicable, details of automation tools used in the process.	5
Data items	10a	List and define all outcomes for which data were sought. Specify whether all results that were compatible with each outcome domain in each study were sought (e.g. for all measures, time points, analyses), and if not, the methods used to decide which results to collect.	4
	10b	List and define all other variables for which data were sought (e.g. participant and intervention characteristics, funding sources). Describe any assumptions made about any missing or unclear information.	7
Study risk of bias assessment	11	Specify the methods used to assess risk of bias in the included studies, including details of the tool(s) used, how many reviewers assessed each study and whether they worked independently, and if applicable, details of automation tools used in the process.	4
Effect measures	12	Specify for each outcome the effect measure(s) (e.g. risk ratio, mean difference) used in the synthesis or presentation of results.	11
Synthesis methods	13a	Describe the processes used to decide which studies were eligible for each synthesis (e.g. tabulating the study intervention characteristics and comparing against the planned groups for each synthesis (item #5)).	5
	13b	Describe any methods required to prepare the data for presentation or synthesis, such as handling of missing summary statistics, or data conversions.	5

Section and Topic	Item #	Checklist item	Location where item is reported
	13c	Describe any methods used to tabulate or visually display results of individual studies and syntheses.	5
	13d	Describe any methods used to synthesize results and provide a rationale for the choice(s). If meta-analysis was performed, describe the model(s), method(s) to identify the presence and extent of statistical heterogeneity, and software package(s) used.	5
	13e	Describe any methods used to explore possible causes of heterogeneity among study results (e.g. subgroup analysis, meta-regression).	5
	13f	Describe any sensitivity analyses conducted to assess robustness of the synthesized results.	5
Reporting bias assessment	14	Describe any methods used to assess risk of bias due to missing results in a synthesis (arising from reporting biases).	N/A
Certainty assessment	15	Describe any methods used to assess certainty (or confidence) in the body of evidence for an outcome.	5
**RESULTS**			
Study selection	16a	Describe the results of the search and selection process, from the number of records identified in the search to the number of studies included in the review, ideally using a flow diagram.	5
	16b	Cite studies that might appear to meet the inclusion criteria, but which were excluded, and explain why they were excluded.	6
Study characteristics	17	Cite each included study and present its characteristics.	6
Risk of bias in studies	18	Present assessments of risk of bias for each included study.	12
Results of individual studies	19	For all outcomes, present, for each study: (a) summary statistics for each group (where appropriate) and (b) an effect estimate and its precision (e.g. confidence/credible interval), ideally using structured tables or plots.	9-12
Results of syntheses	20a	For each synthesis, briefly summarise the characteristics and risk of bias among contributing studies.	13
	20b	Present results of all statistical syntheses conducted. If meta-analysis was done, present for each the summary estimate and its precision (e.g. confidence/credible interval) and measures of statistical heterogeneity. If comparing groups, describe the direction of the effect.	11-16
	20c	Present results of all investigations of possible causes of heterogeneity among study results.	10-11
	20d	Present results of all sensitivity analyses conducted to assess the robustness of the synthesized results.	10-11
Reporting biases	21	Present assessments of risk of bias due to missing results (arising from reporting biases) for each synthesis assessed.	N/A
Certainty of evidence	22	Present assessments of certainty (or confidence) in the body of evidence for each outcome assessed.	11-16
**DISCUSSION**			
Discussion	23a	Provide a general interpretation of the results in the context of other evidence.	17
	23b	Discuss any limitations of the evidence included in the review.	19
	23c	Discuss any limitations of the review processes used.	19

Section and Topic	Item #	Checklist item	Location where item is reported
	23d	Discuss implications of the results for practice, policy, and future research.	19
**OTHER INFORMATION**			
Registration and protocol	24a	Provide registration information for the review, including register name and registration number, or state that the review was not registered.	3
	24b	Indicate where the review protocol can be accessed, or state that a protocol was not prepared.	3
	24c	Describe and explain any amendments to information provided at registration or in the protocol.	N/A
Support	25	Describe sources of financial or non-financial support for the review, and the role of the funders or sponsors in the review.	20
Competing interests	26	Declare any competing interests of review authors.	20
Availability of data, code and other materials	27	Report which of the following are publicly available and where they can be found: template data collection forms; data extracted from included studies; data used for all analyses; analytic code; any other materials used in the review.	20

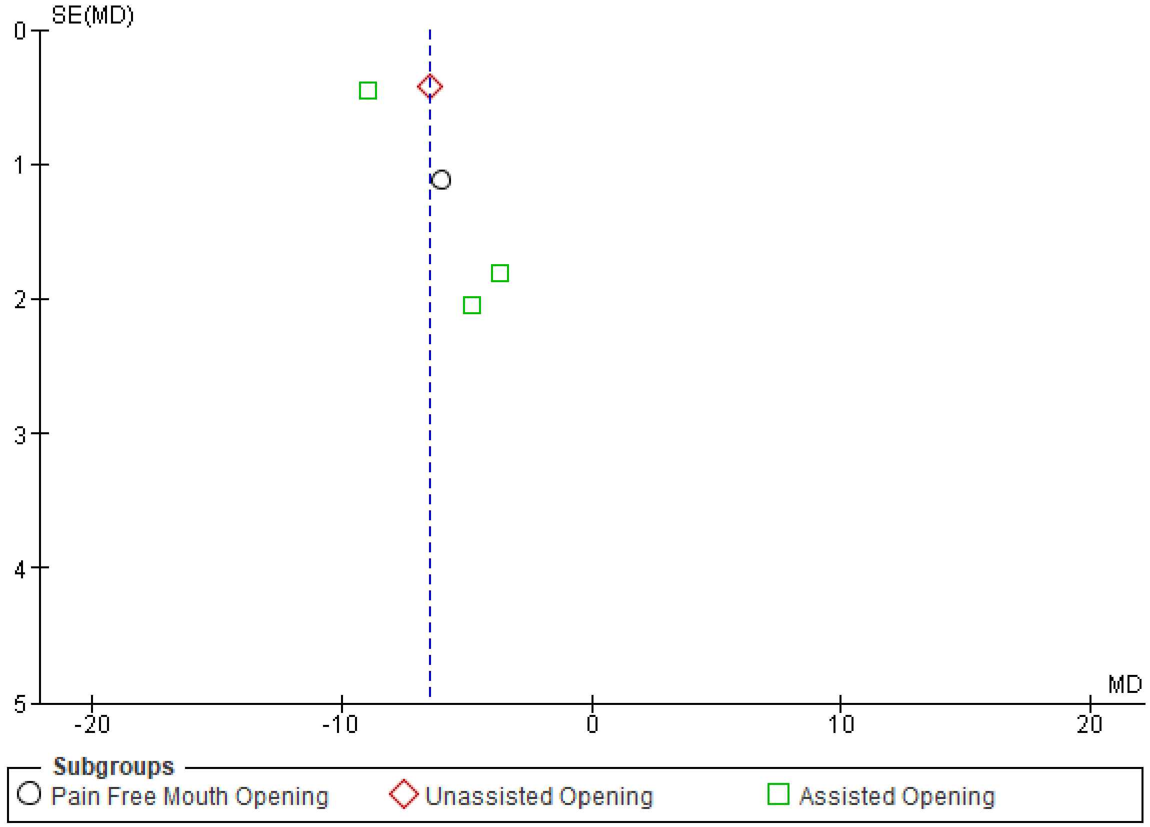

Figure S1. Funnel plot of the meta-analysis on assisted mouth opening. The blue dashed vertical line indicates the pooled (overall) mean difference. SE: Standard Error. MD: Mean Difference
